# Supplementary material for: Rilotumumab Resistance Acquired by Intracrine Hepatocyte Growth Factor Signaling
Source: Cancers (Basel). 2023 Jan 11;15(2):460. doi: 10.3390/cancers15020460 (PMC9857108; doi:10.3390/cancers15020460)
Supplement: Supplementary file 1 [file cancers-15-00460-s001.zip › cancers-2110456-supplementary/Cecchi et al. Raw Blots-1.pdf]

Cecchi et al., Raw Blots, page 1

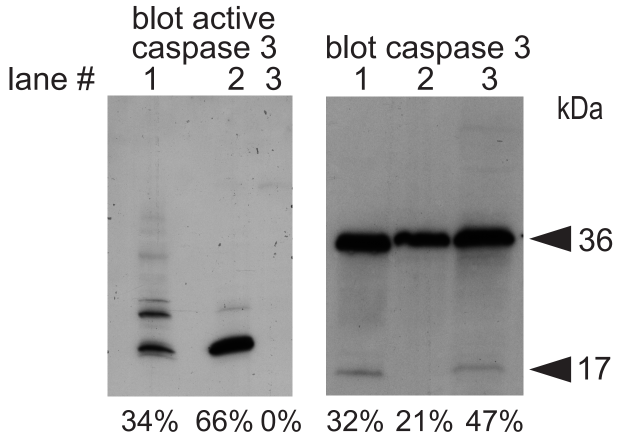

Figure 1C

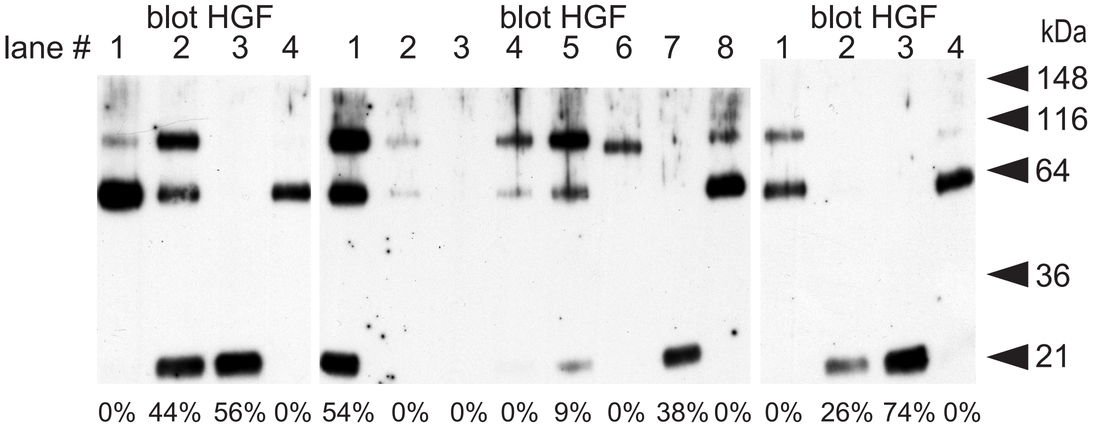

Figure 7C

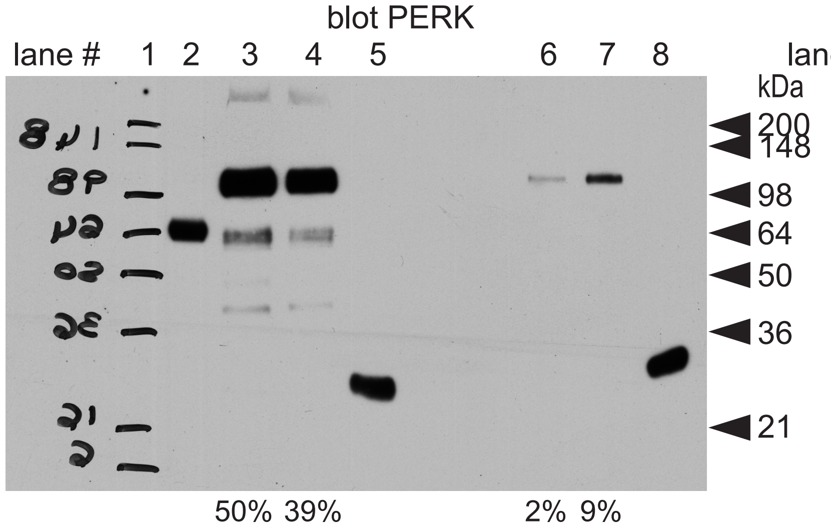

Figure 8A, left

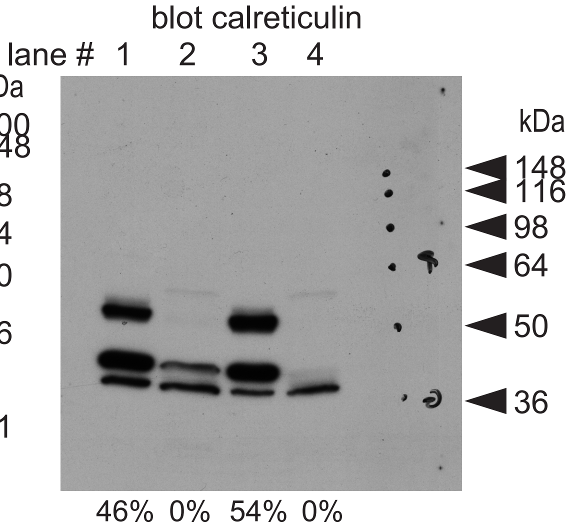

Figure 8A, right

Cecchi et al., Raw Blots, page 2

Figure 8B, upper left
blot p-eIF2-alpha

Figure 8B, lower right
blot eIF2-alpha

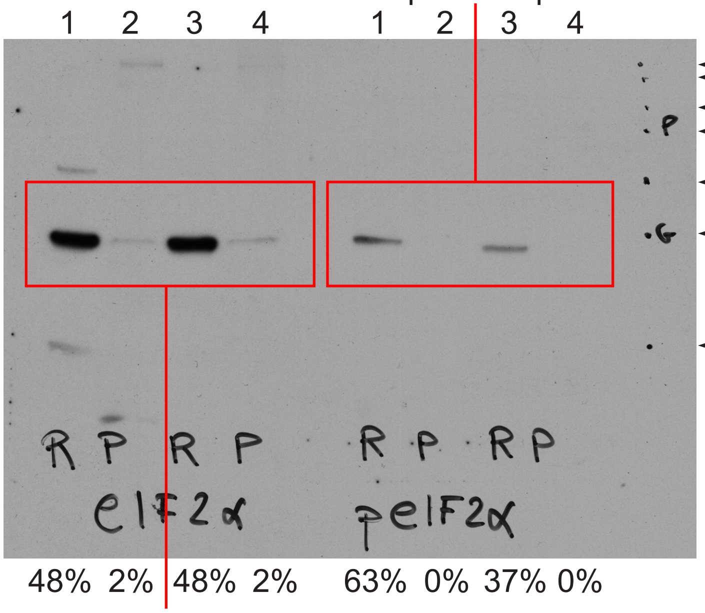

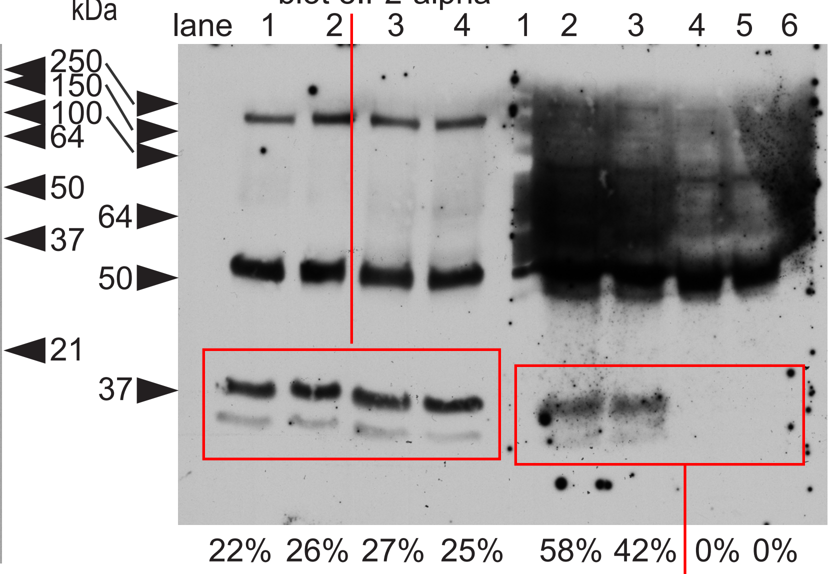

Figure 8B, lower left
blot eIF2-alpha

Figure 8B, upper right
blot p-eIF2-alpha

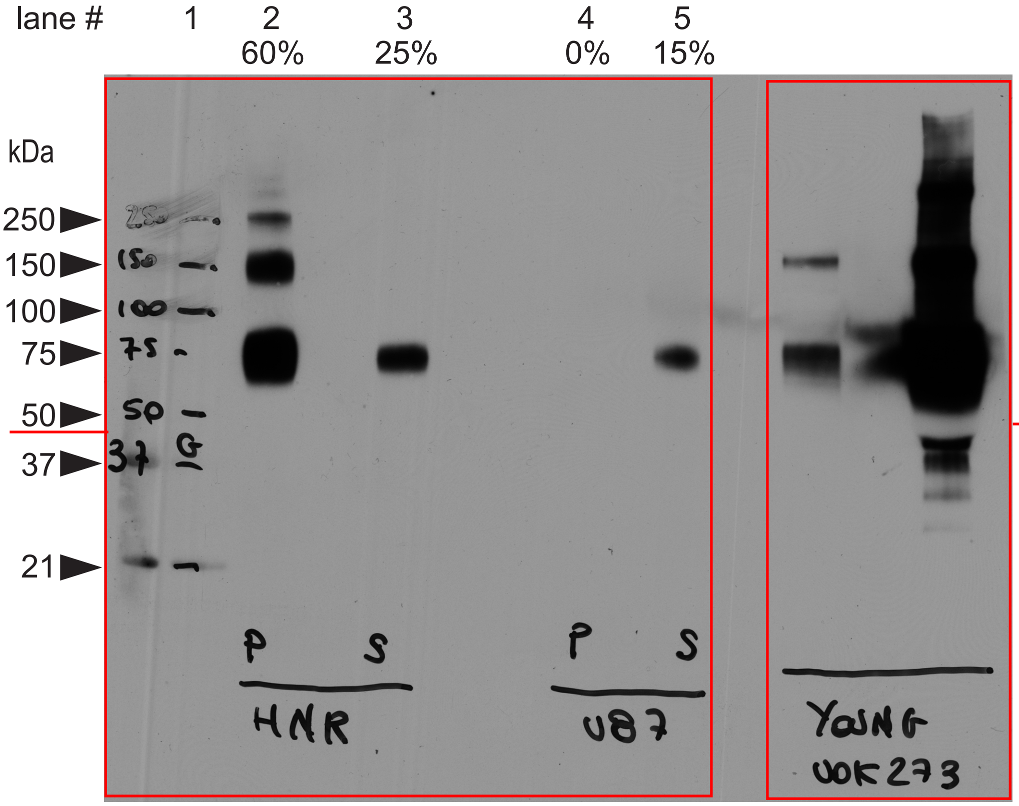

Figure 8C
blot HGF

data for unrelated manuscript
