# Supplementary material for: Rilotumumab Resistance Acquired by Intracrine Hepatocyte Growth Factor Signaling
Source: Cancers (Basel). 2023 Jan 11;15(2):460. doi: 10.3390/cancers15020460 (PMC9857108; doi:10.3390/cancers15020460)
Supplement: Supplementary file 1 [file cancers-15-00460-s001.zip › cancers-2110456-supplementary/Cecchi Table S2.pdf]

INGENUITY
PATHWAY ANALYSIS

Analysis Name: Copy of exp#5846 Parental U87 vs AMG102R U87 -Amgen Confid - 2015-05-18 04:50 PM
Analysis Creation Date: 2015-05-18
Build version: 338830M
Content version: 23814503 (Release Date: 2015-03-22)

Analysis Settings

Reference set: Whole Human Genome Microarray 4x44K v2

Relationship to include: Direct and Indirect

Does not Include Endogenous Chemicals

~~Optional Analyses: My Pathways My List~~

Filter Summary:

Consider only relationships where

confidence = Experimentally Observed

(c) 2000-2015 QIAGEN. All rights reserved.

Top Canonical Pathways

Name	p-value	Overlap
EIF2 Signaling	2.56E-06	55.9 % 95/170
Role of NFAT in Cardiac Hypertrophy	6.63E-06	54.9 % 96/175
Antigen Presentation Pathway	8.67E-06	75.0 % 27/36
Adipogenesis pathway	5.09E-05	56.2 % 68/121
G Protein Signaling Mediated by Tubby	5.51E-05	74.2 % 23/31

Top Upstream Regulators

Upstream Regulator	p-value of overlap	Predicted Activation
TP53	2.28E-22	
camptothecin	1.96E-16	
TGFB1	3.88E-16	
NUPR1	1.14E-12	Activated
ERBB2	1.50E-11	Inhibited

Top Diseases and Bio Functions

Diseases and Disorders

Name	p-value	#Molecules
Cancer	8.35E-04 - 1.20E-13	4515
Organismal Injury and Abnormalities	8.35E-04 - 1.20E-13	4635
Developmental Disorder	8.72E-04 - 1.08E-12	1031
Skeletal and Muscular Disorders	9.08E-04 - 1.08E-12	1047
Gastrointestinal Disease	6.03E-04 - 2.99E-09	2964

Molecular and Cellular Functions

(c) 2000-2015 QIAGEN. All rights reserved.

INGENUITY SYSTEMS

Name	p-value	#Molecules
Cellular Growth and Proliferation	7.12E-04 - 2.79E-25	2146
Cell Death and Survival	9.11E-04 - 1.58E-20	2073
Cellular Development	8.52E-04 - 1.69E-16	2108
Cellular Assembly and Organization	8.39E-04 - 1.06E-14	1181
Cellular Function and Maintenance	8.39E-04 - 1.06E-14	1149

Physiological System Development and Function

Name	p-value	#Molecules
Organismal Survival	4.91E-05 - 1.36E-18	1491
Cardiovascular System Development and Function	8.83E-04 - 4.03E-11	890
Tissue Development	8.83E-04 - 7.98E-11	1663
Hematological System Development and Function	8.19E-04 - 3.56E-09	1007
Embryonic Development	9.11E-04 - 1.17E-08	1368

Top Tox Functions

Assays: Clinical Chemistry and Hematology

Name	p-value	#Molecules
Increased Levels of Red Blood Cells	1.61E-01 - 1.12E-02	47
Decreased Levels of Albumin	6.20E-01 - 2.24E-02	17
Increased Levels of Hematocrit	5.93E-02 - 5.93E-02	41
Increased Levels of Alkaline Phosphatase	3.84E-01 - 6.91E-02	34
Increased Levels of Blood Urea Nitrogen	1.22E-01 - 1.22E-01	8

Cardiotoxicity

(c) 2000-2015 QIAGEN. All rights reserved.

INGENUITY® SYSTEMS

Name	p-value	#Molecules
Cardiac Hypertrophy	1.00E00 - 1.77E-07	193
Cardiac Necrosis/Cell Death	6.20E-01 - 1.64E-03	123
Cardiac Inflammation	6.35E-01 - 2.78E-03	47
Congenital Heart Anomaly	1.00E00 - 3.35E-03	93
Pulmonary Hypertension	6.20E-01 - 4.39E-03	31

Hepatotoxicity

Name	p-value	#Molecules
Hepatocellular Carcinoma	6.35E-01 - 6.45E-05	271
Liver Hyperplasia/Hyperproliferation	6.35E-01 - 6.45E-05	2393
Liver Steatosis	6.20E-01 - 3.89E-04	138
Liver Regeneration	6.20E-01 - 3.90E-03	42
Liver Proliferation	6.20E-01 - 7.22E-03	102

Nephrotoxicity

Name	p-value	#Molecules
Renal Necrosis/Cell Death	5.46E-01 - 1.04E-07	244
Glomerular Injury	6.20E-01 - 4.00E-04	88
Renal Proliferation	6.20E-01 - 2.52E-03	116
Renal Hyperplasia/Hyperproliferation	6.20E-01 - 4.45E-03	15
Renal Hypertrophy	6.20E-01 - 7.49E-03	25

Top Regulator Effect Networks

ID	Regulators	Diseases & Functions	Consistency Score
1	LY6E,mir-199,PADI2,TEAD	cell movement of tumor cell lines,cell viability (+8 more)	11.963

(c) 2000-2015 QIAGEN. All rights reserved.

INGENUITY® SYSTEMS

2	BMI1,CSF2,ERBB2,HGF,IL2,let-7,RAF1,RELA,SP1,STAT1 (+1 more)	migration of squamous cell carcinoma cell lines	9.944
3	HMGB1,KNG1,MEOX2,PADI2	cell movement of cancer cells (+3 more)	9.333
4	HSP90AA1,LY6E,PADI2,TSC22D1	development of body trunk (+4 more)	8.488
5	MEOX2,PLG	fibrogenesis,formation of cytoskeleton (+1 more)	8.004

Top Networks

ID	Associated Network Functions	Score
1	Developmental Disorder, Neurological Disease, Organismal Injury and Abnormalities	21
2	Cell Cycle, Cellular Assembly and Organization, Hereditary Disorder	21
3	Post-Translational Modification, Cell-To-Cell Signaling and Interaction, Cancer	21
4	Molecular Transport, RNA Trafficking, Cell Cycle	21
5	Developmental Disorder, Hereditary Disorder, Metabolic Disease	21

Top Tox Lists

Name	p-value	Overlap	
Renal Necrosis/Cell Death	3.98E-09	51.4 %	244/475
Cardiac Hypertrophy	1.77E-07	51.3 %	193/376
Cardiac Necrosis/Cell Death	6.55E-04	48.4 %	123/254
Biogenesis of Mitochondria	9.71E-04	75.0 %	15/20
Hepatic Fibrosis	1.19E-03	54.2 %	52/96

Top My Lists

Name	p-value	Overlap	
~~cell movement_metastasis 73~~	~~1.52E-03~~	~~56.2 %~~	~~41/73~~

Top Molecules

(c) 2000-2015 QIAGEN. All rights reserved.

INGENUITY SYSTEMS

Fold Change up-regulated

Molecules	Exp. Value	Exp. Chart
**AEBP1**	↑ 100.000	
**AP1M2**	↑ 100.000	
**C15orf48**	↑ 100.000	
**C4BPA**	↑ 100.000	
**CD248**	↑ 100.000	
**CD34**	↑ 100.000	
**CELF2***	↑ 100.000	
**CELF4***	↑ 100.000	
**CHRNA1***	↑ 100.000	
**CXCR3***	↑ 100.000	

Fold Change down-regulated

Molecules	Exp. Value	Exp. Chart
**NDN**	↓ -100.000	
**LMO2**	↓ -100.000	
**FRMD3***	↓ -84.385	
**LUM**	↓ -68.359	
**CSF2**	↓ -66.947	
**IGFBP4**	↓ -48.172	
**COL13A1**	↓ -46.400	
**PTPRU***	↓ -44.695	
**SMAD6**	↓ -43.355	
**LYPD6***	↓ -37.872	

(c) 2000-2015 QIAGEN. All rights reserved.
